# Efficacy and safety of montelukast adjuvant therapy in adults with cough variant asthma: A systematic review and meta‐analysis

**DOI:** 10.1111/crj.13629

**Published:** 2023-05-22

**Authors:** Qian Xu, Tingting Lu, Zhongyang Song, Peng Zhu, Yana Wu, Lumei Zhang, Kehu Yang, Zhiming Zhang

**Affiliations:** ^1^ Clinical College of Traditional Chinese Medicine Gansu University of Chinese Medicine Lanzhou Gansu China; ^2^ Institute of Clinical Research and Evidence‐Based Medicine Gansu Provincial Hospital Lanzhou Gansu China; ^3^ Evidence‐Based Medicine Center, School of Basic Medical Sciences Lanzhou University Lanzhou Gansu China; ^4^ Gansu Provincial Cancer Clinical Research Center of Integrated Traditional Chinese and Western Medicine Affiliated Hospital of Gansu University of Chinese Medicine Lanzhou Gansu China; ^5^ Gansu Provincial Hospital of Traditional Chinese Medicine Lanzhou Gansu China

**Keywords:** cough variant asthma, meta‐analysis, montelukast, randomized controlled trial

## Abstract

**Background:**

Montelukast is a highly selective and specific cysteinyl leukotriene receptor antagonist used in the treatment of asthma. Whether montelukast as adjuvant therapy can significantly and safely treat adults with cough variant asthma (CVA) remains inconclusive.

**Aims:**

This meta‐analysis systematically evaluated the efficacy and safety of montelukast as an adjuvant treatment for adults with CVA.

**Materials and methods:**

Randomized controlled trials (RCTs) on montelukast combined with inhaled corticosteroids (ICS) and long‐acting β2 agonists (LABAs) to treat CVA in adults, from inception to March 6, 2023, were retrieved from the CNKI, Wanfang, VIP, CBM, PubMed, Embase, Cochrane Library, and Web of Science databases and Clinical Trials website. Review Manager (version 5.4) and Stata (version 15.0) were used to conduct the meta‐analysis.

**Results:**

A total of 15 RCTs were ultimately included in the meta‐analysis. It was established that montelukast as adjuvant therapy raised the total effective rate (RR = 1.20, 95% confidence interval [CI] [1.13, 1.27], *P* < 0.01) and improved the FEV1% (SMD = 0.91, 95% CI [0.40, 1.41], *P* < 0.01), PEF% (SMD = 0.63, 95% CI [0.38, 0.88], *P* < 0.01), FEV1 (SMD = 1.15, 95% CI [0.53, 1.77], *P* < 0.01), PEF (SMD = 0.64, 95% CI [0.42, 0.86], *P* < 0.01), and FEV1/FVC% (SMD = 0.76, 95% CI [0.51, 1.01], *P* < 0.01) and reduced the recurrence rate (RR = 0.28, 95% CI [0.15, 0.53], *P* < 0.01). The incidence of adverse reactions was higher in the montelukast auxiliary group compared to the control group but with no statistical difference (RR = 1.32, 95% CI [0.89, 1.96], *P* = 0.17).

**Conclusion:**

Existing evidence indicated that the use of montelukast as an adjuvant therapy had therapeutic efficacy superior to ICS + LABA alone for the treatment of adult patients with CVA. However, further research is needed, especially a combination of high‐quality long‐term prospective studies and carefully designed RCTs.

## Graphical abstract

Whether montelukast as adjuvant therapy can significantly and safely treat adults with cough variant asthma (CVA) remains inconclusive. After a comprehensive retrieve, a total of 15 RCTs were ultimately included in the meta‐analysis. Our results demonstrated that montelukast can play a better curative effect in the adjuvant treatment of adults with CVA.

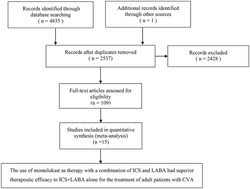

## 1 INTRODUCTION

Cough variant asthma (CVA), which is primarily characterized by cough, is an atypical form of asthma. The cough is irritating and dry, mild during the day, and severe at night. There is airway hyperresponsiveness, but symptoms or signs such as shortness of breath or wheezing are absent, and treatment with anti‐asthmatic drugs is beneficial in patients with CVA.^1^ Epidemiological studies have shown that CVA is the leading cause of chronic cough in China, accounting for about 32–34%.^2^, ^3^ It is the second most common cause after upper airway cough syndrome in Europe and the United States.^4^ Several studies have shown that CVA accounts for about 25–32.6% of chronic cough in adults^2^, ^5^ and about 30–35.7% of patients with CVA eventually develop typical asthma.^6^ The principles for the treatment of CVA are similar to those for asthma.^1^ Treatment with inhaled corticosteroids (ICS) or inhaled corticosteroids combined with long‐acting β2 agonists (ICS + LABA) is recommended for more than 8 weeks.

Treatment with ICS + LABA therapy provides rapid and effective cough relief. However, in patients with poor ICS or severe airway inflammation, a combination of leukotriene receptor antagonists may be used.^7^ Montelukast is a highly selective and specific cysteinyl leukotriene receptor antagonist (CysLTRA). It can relieve bronchospasms and airway mucosal edema by binding to leukotriene receptors, thereby reducing inflammatory cell infiltration and mucus secretion and promoting disease improvement.^8^, ^9^

Clinical studies on montelukast as adjuvant therapy versus ICS + LABA in the treatment of adult CVA have been conducted, but the results are controversial.^10^, ^11^ No meta‐analyses are currently available on this topic. Our study systematically integrated the related randomized controlled trials (RCTs) published at home and abroad on montelukast used as an adjuvant in combination with ICS + LABA for treating adults with CVA.

## 2 MATERIALS AND METHODS

This systematic review was conducted following the Preferred Reporting Items for Systematic Reviews and Meta‐Analysis (PRISMA) statement^12^ and registered in PROSPERO (No. CRD42021289588). Ethical approval and patient consent were not required, as all analyses were based on previously published studies.

### 2.1 Literature search

A comprehensive search of the CNKI, Wanfang, VIP, China Biomedical Literature Database (CBM), PubMed, Embase, Cochrane Library, Web of Science databases, and Clinical Trials (http://www.chictr.org.cn/; https://clinicaltrials.gov) was conducted from database inception to March 6, 2023. The search strategy was as follows: (“cough variant asthma” or “cough variance asthma” or “cough type asthma”) and “montelukast” and (“randomized controlled trial” or “controlled clinical trial” or “random*” or “trial” or “RCT”) (Table S1).

### 2.2 Inclusion and exclusion criteria

The inclusion criteria were as follows: (1) patients aged 18 years or older who were diagnosed with CVA, regardless of gender or race; (2) intervention treatments that included montelukast combined with ICS + LABA versus ICS + LABA for at least 8 weeks; and (3) RCTs, without any restrictions on language or publication type.

The primary outcomes were as follows: (1) the total effective rate and (2) lung function indicators: forced expiratory volume in the first second as a percentage of the predicted value (FEV1%), peak expiratory flow expressed as a percentage of the predicted value (PEF%). The secondary outcomes included (1) lung function indicators: forced expiratory volume in the first second (FEV1), peak expiratory flow (PEF), ratio of the forced expiratory volume in the first second to the forced vital capacity expressed as a percentage (FEV1/FVC%); (2) the recurrence rate; and (3) the incidence of adverse reactions.

Repetitive studies, studies with insufficient data available, animal experiments, literature reviews, meta‐analyses, conference abstracts, case reports, and studies without explicit randomization methods were excluded from our analysis.

### 2.3 Data extraction and quality assessment

Data extraction was conducted by two independent researchers (QX and TTL).^13^, ^14^ It included the first author, publication year, baseline characteristics, intervention measures, treatment course, and outcomes. In case of disagreement, the third author (ZYS) was consulted to reach an agreement.^15^, ^16^, ^17^

The methodological quality assessment of included trials was independently evaluated by the two authors (QX and TTL) based on the Cochrane Collaboration risk of bias assessment tool (ROB).^18^ The evaluation domains were as follows: random sequence generation, allocation concealment, blinding of participants and personnel, blinding of outcome assessment, incomplete outcome data, selective reporting, and other biases. Each domain was rated as low, unclear, or high risk of bias.

### 2.4 Statistical analysis

Dichotomous outcomes are expressed as the relative risk (RR) with a 95% confidence interval (CI), while continuous outcomes are presented by the standardized mean difference (SMD) with 95% CI. Heterogeneity was evaluated using the Chi‐square and *I* ^2^ statistics. Only when the *P* value was >0.1 or *I* ^2^ was ≤50% was the fixed‐effects model performed to combine effect sizes. In all other cases, the random‐effects model was adopted. Subgroup analysis was conducted according to cough symptoms and recurrence and cough symptoms and bronchial provocation tests in the studies, which were included in the analysis. Sensitivity analysis was also performed to further identify potential sources of heterogeneity. Data analysis was performed by Review Manager (version 5.4), and Stata (version 15.0) was applied to detect publication bias. A *P* value of less than 0.05 was considered to indicate a statistically significant difference.

## 3 RESULTS

### 3.1 Literature search

**FIGURE 1:**
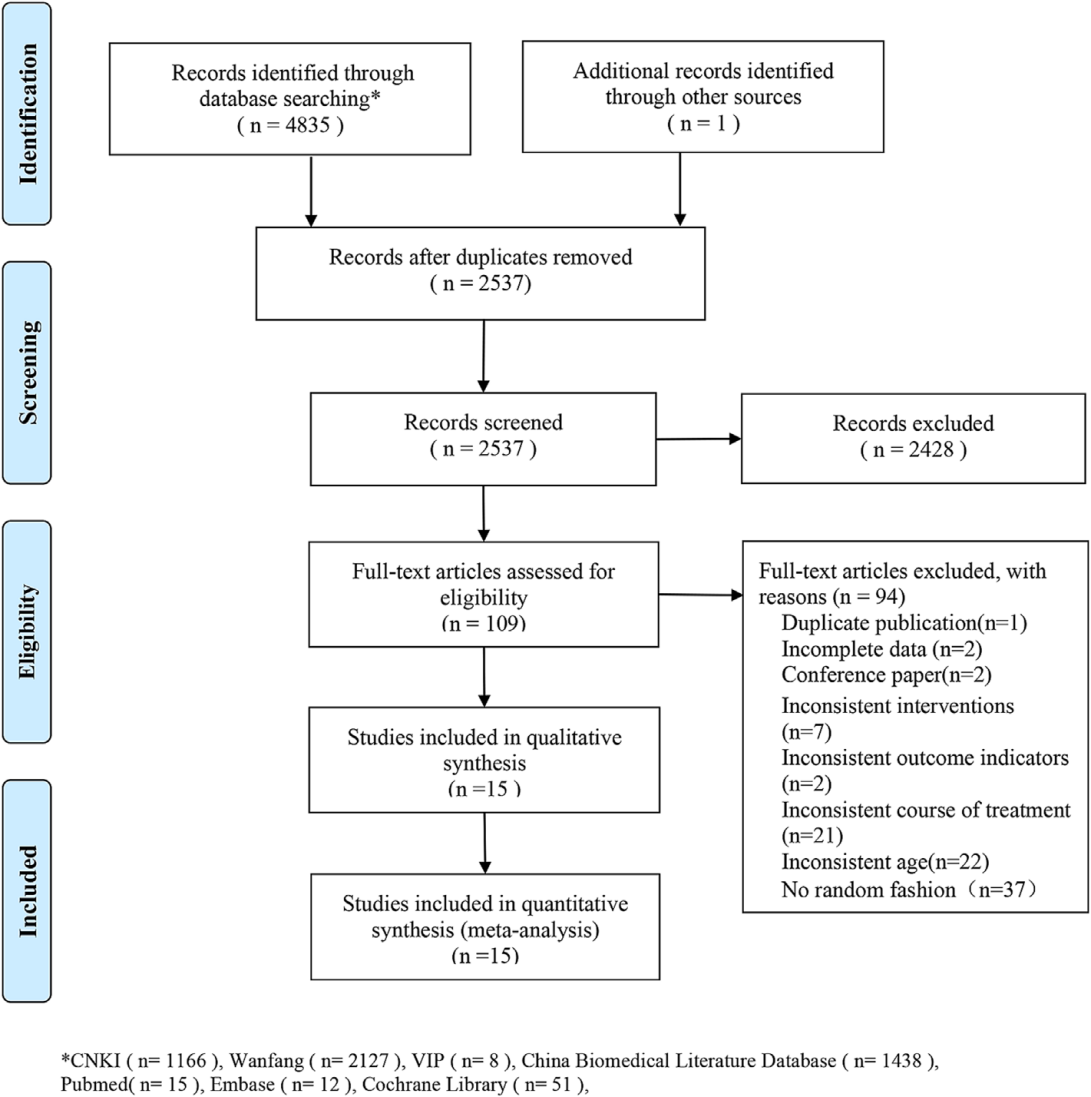
PRISMA flow diagram of the study selection process.

A total of 4836 studies were screened. After removing duplicates, 2537 remained for screening of the titles and abstracts, and the full text of 109 studies was read. Eventually, 15 studies,^19^, ^20^, ^21^, ^22^, ^23^, ^24^, ^25^, ^26^, ^27^, ^28^, ^29^, ^30^, ^31^, ^32^, ^33^ which were all conducted in China, were included according to the eligibility criteria (Figure 1).

### 3.2 Study characteristics

Fifteen RCTs,^19^, ^20^, ^21^, ^22^, ^23^, ^24^, ^25^, ^26^, ^27^, ^28^, ^29^, ^30^, ^31^, ^32^, ^33^ which were published from 2014 to 2022, with a total of 1314 participants, were included in the analysis. Montelukast combined with budesonide formoterol was used for treatment in seven studies,^19^, ^21^, ^23^, ^24^, ^29^, ^32^, ^33^ and montelukast combined with salmeterol‐ticasone was used in the remaining studies.^20^, ^22^, ^25^, ^26^, ^27^, ^28^, ^30^, ^31^ The total course of treatment was 8 weeks in 10 of the studies^19^, ^20^, ^21^, ^22^, ^26^, ^27^, ^29^, ^30^, ^32^, ^33^ and 12 weeks in the other studies.^23^, ^24^, ^25^, ^28^, ^31^ The main characteristics of the included studies are summarized in Table 1.

**TABLE 1:** Characteristics of the included studies

Study ID	Sample size (*n*)		Gender (male/female)		Age (mean ± standard deviation)		Course of disease (mean ± standard deviation)		Intervention		Course of treatment (w)	Outcomes
	Treatment	Contr‐ol	Treatment	Contr‐ol	Treatment	Control	Treatment	Control	Treatment	Control		
Chen/2017	40	40	19/21	17/23	32.9 ± 16.2	32.3 ± 16.8	3.8 ± 3.5 years	3.9 ± 4.1 years	Budesonide formoterol+ montelukast	Budesonide formoterol	8	1
Feng/2015	49	49	24/25	22/27	45.7 ± 3.8	46.4 ± 3.5	Not described		Salmeterol ticasone+ montelukast	Salmeterol ticasone	8	1, 7
Gao/2022	34	34	14/20	16/18	40.24 ± 6.61	40.89 ± 6.68	6.21 ± 0.55 months	6.08 ± 0.53 months	Budesonide formoterol+ montelukast	Budesonide formoterol	8	1, 4, 5, 8
Guo/2019	52	52	29/23	27/25	34.78 ± 5.36	34.93 ± 5.24	15.36 ± 3.49 months	15.19 ± 3.57 months	Salmeterol ticasone+ montelukast	Salmeterol ticasone	8	4, 8
Li/2018	47	47	26/21	25/22	51.14 ± 11.05	50.83 ± 10.95	6.48 ± 2.37 years	6.26 ± 2.28 years	Budesonide formoterol+ montelukast	Budesonide formoterol	12	1
Liu/2014	49	48	52/45		65.1 ± 3.8		7.05 ± 0.75 years		Budesonide formoterol+ montelukast	Budesonide formoterol	12	4, 5, 6
Su/2021	62	62	33/29	34/28	68.41 ± 4.42	67.32 ± 4.54	5.61 ± 1.13 months	5.53 ± 1.16 months	Salmeterol ticasone+ montelukast	Salmeterol ticasone	12	1, 7, 8
Wang/2016	48	48	22/26	21/27	35.8 ± 8.6	36.3 ± 8.3	16.4 ± 5.7 months	16.1 ± 5.4 months	Salmeterol ticasone+ montelukast	Salmeterol ticasone	8	1, 2, 3, 4, 6, 7, 8
Wu/2012	36	36	25/11	24/12	38.5 ± 11.8	37.8 ± 12.2	11.8 ± 5.7 months	12.6 ± 6.1 months	Salmeterol ticasone+ montelukast	Salmeterol ticasone	8	1, 2, 3, 8
Ye/2018	36	39	20/16	21/18	33.1 ± 8.6	33.5 ± 7.9	17.1 ± 4.2 months	16.8 ± 3.6 months	Salmeterol ticasone+ montelukast	Salmeterol ticasone	12	1, 4, 6, 7, 8
Ye/2019	53	53	33/20	30/23	38.63 ± 4.61	37.93 ± 4.17	5.13 ± 0.78 months	5.20 ± 1.10 months	Budesonide formoterol+ montelukast	Budesonide formoterol	8	1, 4, 5, 8
Zhang/2015	49	49	20/29	22/27	38.3 ± 8.2	38.8 ± 7.8	15.5 ± 6.6 months	15.6 ± 6.7 months	Salmeterol ticasone+ montelukast	Salmeterol ticasone	8	1, 8
Zhang/2022	25	25	15/10	17/8	65.85 ± 2.44	65.56 ± 2.37	Not described		Salmeterol ticasone+ montelukast	Salmeterol ticasone	12	1, 8
Zhou/2021	30	30	17/13	15/15	37.35 ± 9.31	38.27 ± 9.65	12.82 ± 4.84 weeks	13.34 ± 4.90 weeks	Budesonide formoterol+ montelukast	Budesonide formoterol	8	4, 5, 8
Zhu/2018	46	46	21/25	19/27	41.36 ± 9.49	42.04 ± 9.21	3.16 ± 0.80 months	2.97 ± 0.93 months	Budesonide formoterol+ montelukast	Budesonide formoterol	8	2, 3, 8

### 3.3 Quality assessment

**FIGURE 2:**
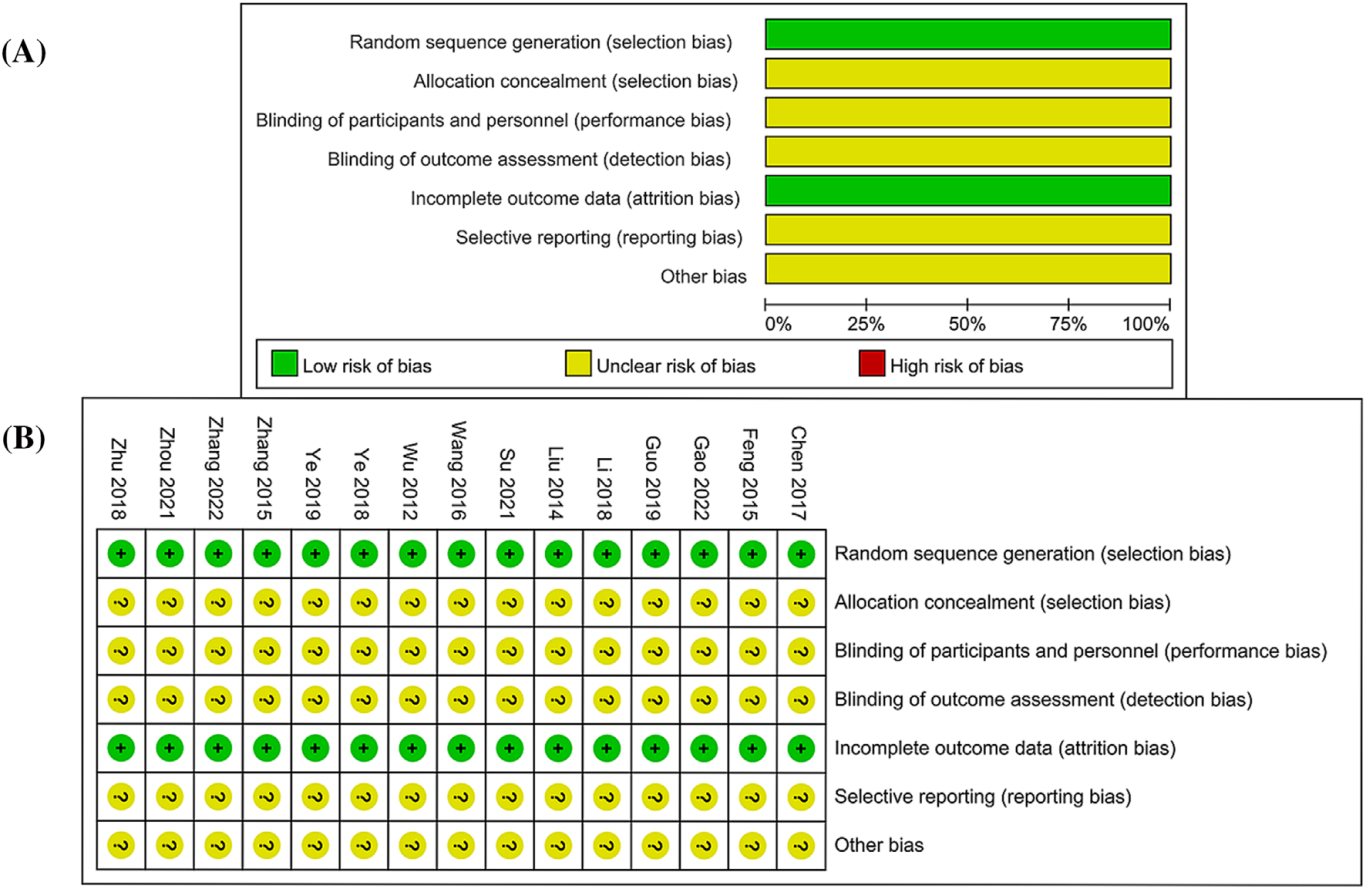
Assessment of risk of bias: (A) risk of bias graph; (B) risk of bias summary.

All included studies were evaluated according to the Cochrane Collaboration ROB tool. Random sequence generation was explicit in all studies, of which one study used random envelopes^19^ while the others used a random number table.^20^, ^21^, ^22^, ^23^, ^24^, ^25^, ^26^, ^27^, ^28^, ^29^, ^30^, ^31^, ^32^, ^33^ The assessment results were unclear since neither allocation concealment nor the blinding method was mentioned in the overall studies.^19^, ^20^, ^21^, ^22^, ^23^, ^24^, ^25^, ^26^, ^27^, ^28^, ^29^, ^30^, ^31^, ^32^, ^33^ All studies^19^, ^20^, ^21^, ^22^, ^23^, ^24^, ^25^, ^26^, ^27^, ^28^, ^29^, ^30^, ^31^, ^32^, ^33^ had complete outcome data, but there was no detailed information for evaluating whether they had selective outcome reporting or other sources of risk of bias (Figure 2).

### 3.4 Primary outcomes

#### 3.4.1 The total effective rate

**FIGURE 3:**
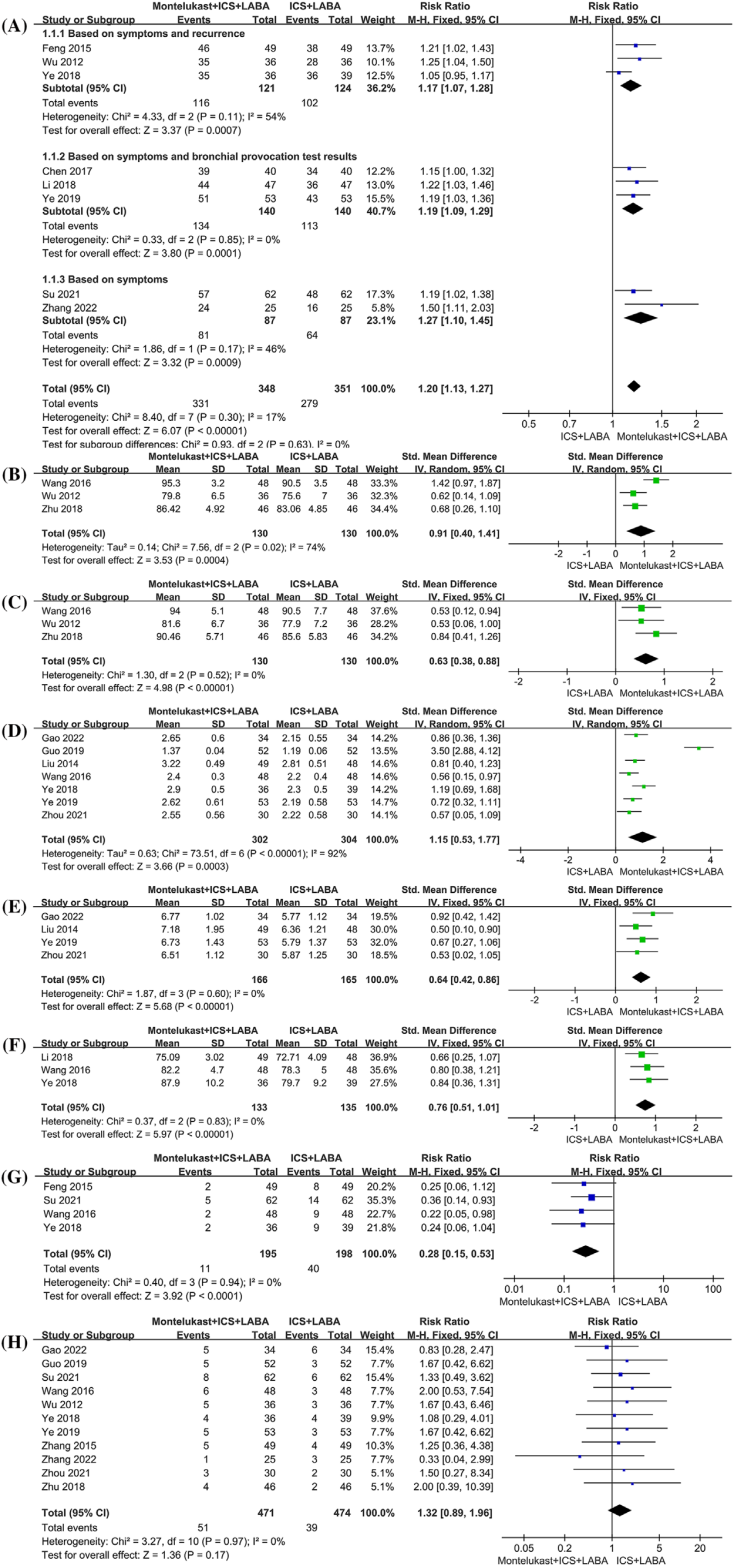
Forest plots with montelukast adjuvant group versus the control group: (A) total effective rate; (B) FEV1%; (C) PEF%; (D) FEV1; (E) PEF; (F) FEV1/FVC%; (G) recurrence rate; (H) incidence of adverse reactions.

A total of 11 RCTs^19^, ^20^, ^21^, ^23^, ^25^, ^26^, ^27^, ^28^, ^29^, ^30^, ^31^ mentioned the total effective rate. Among them, Gao^21^ used lung function improvement as the evaluation criterion, Wang and Li^26^ used inconsistent cough symptom scores as evaluation criteria, and Zhang^30^ proposed evaluation criteria based on whether cough symptoms were relieved following 2 weeks of treatment. Thus, the effect sizes of the above three studies were not combined. No heterogeneity was seen among the remaining eight studies^19^, ^20^, ^23^, ^25^, ^27^, ^28^, ^29^, ^31^ that reported the total effective rate (*P* = 0.30, *I* ^2^ = 17%). Meta‐analysis using the fixed‐effects model established that the total effective rate of montelukast adjuvant treatment was higher than that in the control group (RR = 1.20, 95% CI [1.13, 1.27], *P* < 0.01). Among these eight studies,^19^, ^20^, ^23^, ^25^, ^27^, ^28^, ^29^, ^31^ there were three different evaluation criteria for the total effective rate. Three studies^20^, ^27^, ^28^ with moderate heterogeneity (*I* ^2^ = 54%) used cough symptoms and recurrence as evaluation criteria. The results of this subgroup with the fixed‐effects model apparently confirmed that the total effective rate was higher in the montelukast adjuvant group compared to the control group (RR = 1.17, 95% CI [1.07, 1.28], *P* < 0.01). After excluding the study by Ye and Feng,^28^ the conclusion that montelukast improved the total effective rate remained unchanged (RR = 1.23, 95% CI [1.08, 1.39], *P* < 0.01), but heterogeneity was decreased (*P* = 0.80, *I* ^2^ = 0%). Three studies^19^, ^23^, ^29^ with no heterogeneity (*P* = 0.85, *I* ^2^ = 0%) were based on cough symptoms and bronchial provocation test results. The fixed‐effects meta‐analysis demonstrated a statistical difference in favor of the montelukast adjuvant group (RR = 1.19, 95% CI [1.09, 1.29], *P* < 0.01). The other two studies^25^, ^31^ based on unified cough symptom scores had mild heterogeneity (*P* = 0.17, *I* ^2^ = 46%). Meta‐analysis using the fixed‐effects model showed that the montelukast adjuvant treatment group had a higher total effective rate (RR = 1.27, 95% CI [1.10, 1.45], *P* < 0.01) (Figure 3A).

#### 3.4.2 The lung function indicators: FEV1% and PEF%

##### FEV1%

A noticeable difference in favor of the montelukast adjuvant group (SMD = 0.91, 95% CI [0.40, 1.41], *P* < 0.01) was observed using the random‐effects model in three studies^26^, ^27^, ^33^ reporting FEV1% with moderate heterogeneity (*P* = 0.02, *I* ^2^ = 74%) (Figure 3B). Due to the moderate heterogeneity of the results, sensitivity analysis found that the conclusion that montelukast could improve FEV1% after excluding Wang and Li's study^26^ remained unchanged, and the difference between groups was statistically significant (SMD = 0.65, 95% CI [0.34, 0.97], *P* < 0.01). However, the heterogeneity was significantly decreased (*P* = 0.84, *I* ^2^ = 0%).

##### PEF%

The PEF% index reported in three studies^26^, ^27^, ^33^ without heterogeneity (*P* = 0.52, *I* ^2^ = 0%) was combined by the fixed‐effects model. An obvious difference in favor of the montelukast adjuvant group was demonstrated (SMD = 0.63, 95% CI [0.38, 0.88], *P* < 0.01) (Figure 3C).

### 3.5 Secondary outcomes

#### 3.5.1 Lung function indicators: FEV1, PEF, and FEV1/FVC%

##### FEV1

Seven studies^21^, ^22^, ^24^, ^26^, ^28^, ^29^, ^32^ reporting FEV1 showed high heterogeneity (*P* < 0.01, *I* ^2^ = 92%). The random‐effects meta‐analysis demonstrated that the FEV1 was improved by montelukast adjuvant therapy with a statistical difference (SMD = 1.15, 95% CI [0.53, 1.77], *P* < 0.01) (Figure 3D). Due to the high heterogeneity, sensitivity analysis was conducted by eliminating the studies one by one. After eliminating one study,^22^ the conclusion that montelukast improved FEV1 remained unchanged (SMD = 0.77, 95% CI [0.59, 0.95], *P* < 0.01). However, heterogeneity decreased (*P* = 0.47, *I* ^2^ = 0%).

##### PEF

Four studies^21^, ^24^, ^29^, ^32^ reported the PEF index with no heterogeneity (*P* = 0.60, *I* ^2^ = 0%). The results using the fixed‐effects model demonstrated an obvious difference in favor of the montelukast adjuvant group (SMD = 0.64, 95% CI [0.42, 0.86], *P* < 0.01) (Figure 3E).

##### FEV1/FVC%

An evident difference in favor of the montelukast adjuvant group was confirmed in three studies,^24^, ^26^, ^28^ which recorded the FEV1/FVC% index (SMD = 0.76, 95% CI [0.51, 1.01], *P* < 0.01). The fixed‐effects model was performed because no heterogeneity was seen (*P* = 0.83, *I* ^2^ = 0%) (Figure 3F).

#### 3.5.2 Recurrence rate

Four studies^20^, ^25^, ^26^, ^28^ reported the recurrence rate after 6 months of follow‐up. No heterogeneity was observed (*P* = 0.94, *I* ^2^ = 0%). The fixed‐effect model was then conducted and indicated that montelukast adjuvant treatment reduced the recurrence rate (RR = 0.28, 95% CI [0.15, 0.53], *P* < 0.01) (Figure 3G).

#### 3.5.3 The incidence of adverse reactions

Eleven studies^21^, ^22^, ^25^, ^26^, ^27^, ^28^, ^29^, ^30^, ^31^, ^32^, ^33^ reported the incidence of adverse events, and no apparent heterogeneity was obtained among them (*P* = 0.97, *I* ^2^ = 0%). Although a higher incidence of adverse reactions was seen in the montelukast adjuvant group, the meta‐analysis with the fixed‐effects model showed no statistical difference compared to the control group (RR = 1.32, 95% CI [0.89, 1.96], *P* = 0.17) (Figure 3H).

### 3.6 Publication bias

**FIGURE 4:**
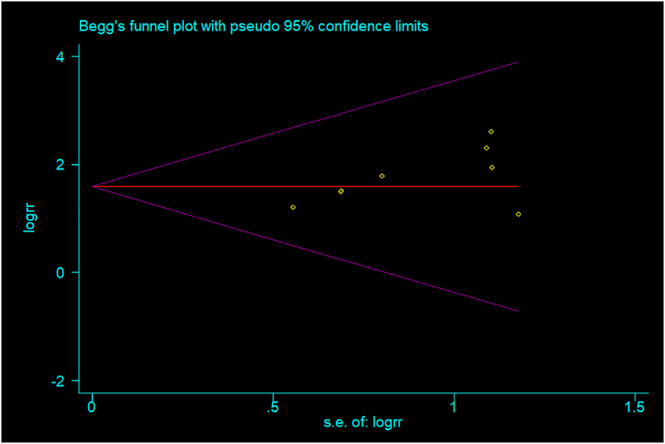
Publication bias funnel chart.

The result of Begg's test showed that the total effective rate of this study had no publication bias (*P* = 0.079) (Figure 4), which to some extent supported the reliability of the research results.

## 4 DISCUSSION

CVA is the leading cause of chronic cough in China and is the second most common cause of chronic cough in European and American countries. It also accounts for about 25–32.6% of chronic cough in adults. Irritant dry cough is the main clinical symptom of CVA. A positive bronchial provocation test is still essentially the gold standard for diagnosis, and effective anti‐asthma treatment is a necessary diagnostic condition.^34^ It is worth noting that uncontrolled and recurrent CVA may lead to the development of classic asthma,^35^ which in turn further leads to worsening lung function. Hence, early intervention and standardized CVA treatment are crucial. Domestic and international guidelines^1^, ^4^ recommend treatment with ICS or ICS + LABA for the management of CVA. Leukotriene receptor antagonists can be used in patients who cannot use hormones or whose hormonal therapy is ineffective.

Currently, airway inflammation, remodeling, and airway hyperresponsiveness are widely recognized to play a critical role in the pathogenesis of CVA.^36^ Studies found that although the clinical symptoms of CVA patients were milder compared to those with typical asthma, increases in the degree of eosinophilia in the central airway mucosal tissue biopsy and bronchoalveolar lavage fluid were the same.^37^ Cysteinyl leukotrienes (CysLTs), which are closely related to airway inflammation of CVA, are mainly produced by eosinophils. CysLTs mainly include LTC4, LTD4, LTE4, and LTF4, which can lead to bronchial smooth muscle contraction, mucosal edema, and mucus secretion. Montelukast is a cysteinyl leukotriene receptor (CysLTR) antagonist, which can selectively block the CysLT1R to inhibit inflammation of the airway. Leukotrienes are also involved in CVA airway remodeling. Studies suggested that LTC4 and LTD4 are the primary leukotrienes that mediate airway remodeling. Both of them have a high affinity for CysLT1R.^38^ Montelukast can inhibit airway remodeling by blocking CysLT1R. Some scholars^39^ compared the LTC4 concentration and LTC4/PGE2 ratio in induced sputum of CVA, typical asthma, and eosinophilic bronchitis and suggested that high LTC4 concentrations and high LTC4/PGE2 ratios are the inflammatory basis of airway hyperresponsiveness. Montelukast can inhibit airway hyperresponsiveness by preventing LTC4 from binding to CysLT1R.

Fifteen studies were eventually included in our analysis. All these studies were based on the use of ICS + LABA combined with montelukast for adjuvant treatment of CVA in adults for at least 8 weeks. Meta‐analysis suggested that montelukast adjuvant therapy increased the total response rate, improved lung function indicators (FEV1%, PEF%, FEV1, PEF, and FEV1/FVC%), and reduced the recurrence rate. Although the incidence of adverse reactions in the montelukast adjuvant group was higher, they were alleviated spontaneously or after symptomatic treatment. There was moderate heterogeneity when analyzing the total effective rate based on cough symptoms combined with recurrence. When Ye and Feng's study was excluded, heterogeneity was significantly decreased. Studying the original text of Ye and Feng^28^ found that the patients were 19–57 years old, the course of the disease was 3–23 months, and the montelukast treatment cycle reached 12 weeks. Due to the moderate heterogeneity in FEV1%, sensitivity analysis was conducted, which indicated that the study by Wang and Li^26^ may have been the cause of the heterogeneity. In their study, patients were aged 20–55 and had a disease course of 2–25 months. All the patients received 10 mg of montelukast before bedtime for 8 weeks. In the process of analyzing FEV1 attributed to the high heterogeneity, a sensitivity analysis was also carried out, which found that heterogeneity decreased after removing the research by Guo.^22^ Analysis of the original study found that patients in the study by Guo^22^ were 19–60 years old and had a disease duration of 2–24 months. Montelukast was given at 10 mg once a day for an unlimited medication time, and the treatment course was 8 weeks. Considering the actual situation, all the above had the potential to produce heterogeneity.

A meta‐analysis confirmed that montelukast combined with budesonide significantly increased the total effective rate; improved FEV1, FEV1%, and PEF; and reduced the recurrence rate in the treatment of children with CVA compared to budesonide alone.^40^ A study by Feng et al.^41^ found that a treatment regimen of montelukast given with a combination of salmeterol‐ticasone had a higher total response rate and apparent efficiency, shorter cough duration, and lower recurrence rate without limitations on the age of CVA patients compared to when salmeterol‐ticasone was given alone. The above studies were consistent with our results. In addition, our study limited the inclusion of adults with CVA, and the treatment of montelukast combined with ICS + LABA was used for at least 8 weeks. The results confirmed that the standardized treatment of montelukast was effective for adult CVA patients. Furthermore, reliable indicators like PEF% and FEV1/FVC%, which also reflect lung function, were added to the outcome and showed improvement, further verifying the effectiveness of montelukast in improving lung function.

Our study had several strengths. First, this is the first study, to the best of our knowledge, that conducted a systematic review and meta‐analysis of montelukast as an adjunctive treatment for adults with cough variant asthma. Second, data screening, extraction, and quality evaluation were all individually completed by two researchers to ensure the reliability of the data. Additionally, subgroup analysis was also carried out to further investigate the source of heterogeneity. Our study also had some limitations. First, blinding was not mentioned in all included studies, which in turn might have led to implementation bias. Second, although the dose of montelukast in all included studies was 10 mg, the frequency and duration of use were not consistent across all the studies. Third, the small number of included studies and small sample size might have impacted the reliability of the results of this systematic review. Therefore, future studies should clarify the implementation of blinding methods when conducting relevant studies, standardize the application of montelukast according to guidelines, and conduct large‐scale multicenter RCTs to improve the reliability of the research results.

Existing evidence indicated that the use of montelukast as an adjuvant therapy had therapeutic efficacy superior to that of ICS + LABA alone for the treatment of adult patients with CVA. However, based on the above limitations, further research is needed, especially a combination of high‐quality long‐term prospective studies and carefully designed RCTs.

## AUTHOR CONTRIBUTIONS

Kehu Yang and Zhiming Zhang designed the study. Qian Xu and Tingting Lu were in charge of literature search and data analysis. Qian Xu and Tingting Lu were responsible for the initial manuscript. Zhongyang Song, Peng Zhu, Yana Wu, and Lumei Zhang contributed to the collection of data. All authors commit to be responsible for all aspects of the work.

## CONFLICT OF INTEREST STATEMENT

All authors declare no conflict of interest.

## ETHICS STATEMENT

All analyses in this study were based on previously published results and did not require ethical approval or patient consent.

## Supporting information


**Table S1.** Search Strategy in PubMed

## Data Availability

The data that support the findings of this study are available from the corresponding author upon reasonable request.
