# Supplementary material for: Efficacy and safety of montelukast adjuvant therapy in adults with cough variant asthma: A systematic review and meta‐analysis
Source: Clin Respir J. 2023 May 22;17(10):986–97. doi: 10.1111/crj.13629 (PMC10543051; doi:10.1111/crj.13629)
Supplement: Supplementary file 1 — Table S1. Search Strategy in PubMed [file CRJ-17-986-s001.docx]

Table S1 Search Strategy in PubMed

| Search number | Query | Search Details | Results |
| --- | --- | --- | --- |
| 8 | ((#1) AND (#4)) AND (#7) | ("cough type asthma"[Title/Abstract] OR ((("cough"[MeSH Terms] OR "cough"[All Fields] OR "coughing"[All Fields] OR "coughs"[All Fields] OR "coughed"[All Fields]) AND ("variance"[All Fields] OR "variances"[All Fields])) AND "asthma"[Title/Abstract]) OR "cough variant asthma"[Title/Abstract]) AND ("Montelukast"[Supplementary Concept] OR ("MK-0476"[Title/Abstract] OR "MK-0476"[Title/Abstract] OR "Singulair"[Title/Abstract] OR "montelukast sodium"[Title/Abstract])) AND ("random*"[Title/Abstract] OR "trial*"[Title/Abstract] OR ("Randomized Controlled Trial"[Publication Type] OR "Randomized Controlled trials as Topic"[MeSH Terms] OR "Random Allocation"[MeSH Terms] OR "Controlled Clinical Trial"[Publication Type] OR "Controlled Clinical Trials as Topic"[MeSH Terms])) | 15 |
| 7 | (#5) OR (#6) | "random*"[Title/Abstract] OR "trial*"[Title/Abstract] OR "Randomized Controlled Trial"[Publication Type] OR "Randomized Controlled trials as Topic"[MeSH Terms] OR "Random Allocation"[MeSH Terms] OR "Controlled Clinical Trial"[Publication Type] OR "Controlled Clinical Trials as Topic"[MeSH Terms] | 2,369,227 |
| 6 | Random* [Title/Abstract] OR Trial* [Title/Abstract] | "random*"[Title/Abstract] OR "trial*"[Title/Abstract] | 2,102,951 |
| 5 | "Randomized Controlled Trial" [Publication Type] OR "Randomized Controlled trials as Topic" [Mesh] OR "Random Allocation" [Mesh] OR "Controlled Clinical Trial" [Publication Type] OR "Controlled Clinical Trials as Topic " [Mesh] | "Randomized Controlled Trial"[Publication Type] OR "Randomized Controlled trials as Topic"[MeSH Terms] OR "Random Allocation"[MeSH Terms] OR "Controlled Clinical Trial"[Publication Type] OR "Controlled Clinical Trials as Topic"[MeSH Terms] | 926,702 |
| 4 | (#2) OR (#3) | "Montelukast"[Supplementary Concept] OR "MK-0476"[Title/Abstract] OR "MK-0476"[Title/Abstract] OR "Singulair"[Title/Abstract] OR "montelukast sodium"[Title/Abstract] | 2,016 |
| 3 | (((MK 0476[Title/Abstract]) OR (MK-0476[Title/Abstract])) OR (Singulair[Title/Abstract])) OR (Montelukast sodium[Title/Abstract]) | "MK-0476"[Title/Abstract] OR "MK-0476"[Title/Abstract] OR "Singulair"[Title/Abstract] OR "montelukast sodium"[Title/Abstract] | 293 |
| 2 | "montelukast" [Supplementary Concept] | "montelukast"[Supplementary Concept] | 1,922 |
| 1 | ((cough type asthma[Title/Abstract]) OR (cough variance asthma[Title/Abstract])) OR (cough variant asthma[Title/Abstract]) | "cough type asthma"[Title/Abstract] OR ((("cough"[MeSH Terms] OR "cough"[All Fields] OR "coughing"[All Fields] OR "coughs"[All Fields] OR "coughed"[All Fields]) AND ("variance"[All Fields] OR "variances"[All Fields])) AND "asthma"[Title/Abstract]) OR "cough variant asthma"[Title/Abstract] | 549 |
